# Neu1 inhibition restrains BCoV replication and modulates ZBP1-dependent PANoptosis

**DOI:** 10.1186/s13567-026-01729-7

**Published:** 2026-04-21

**Authors:** Haoyuan Ma, Jiawei Zhao, Kai Yu, Siqi Zhang, Jilong Liu, Hao Yu, Shuoning Cao, Jianyou Jin, Shujiang Xue, Qiang Li, Zhiqiang Xu, Shengwei Ji, Chenghui Li, Xinpeng Ji, Zheng Sun, Jingrui Hao, Jialiang Xie, Rumeng Tian, Xifeng Zhang, Rui Du, Xu Gao

**Affiliations:** Laboratory for Animal Molecular Virology, Department of Veterinary Medicine, College of Agriculture, Yanbian University, Yanji, 133002 China

**Keywords:** *Betacoronavirus*, bovine coronavirus, *Neu1*, *ZBP1*, PANoptosis

## Abstract

As a member of the *Betacoronavirus* genus, bovine coronavirus (BCoV) is a major etiological agent in cattle, causing diarrhea, fever, and reduced milk production, thereby imposing a substantial economic burden on the livestock industry. However, the molecular mechanisms underlying BCoV pathogenesis remain incompletely defined. In this study, we report that BCoV infection of Madin–Darby bovine kidney (MDBK) cells activates ZBP1–PANoptosome-associated signaling and induces the host sialidase neuraminidase 1 (Neu1). Functional studies using CRISPR–Cas9-mediated knockdown revealed that *Neu1* depletion attenuated ZBP1–PANoptosome activation and reduced viral replication in vitro. Co-immunoprecipitation and GST pull-down assays, together with molecular docking and molecular dynamics analyses, supported a physical association between Neu1 and ZBP1, suggesting a potentially stable interaction interface. In vivo, oral inoculation of BALB/c mice with BCoV resulted in increased Neu1 and ZBP1 signals with partial colocalization in brain and colonic tissues, accompanied by decreased serum sialic acid levels and elevated interleukin-1 beta (IL-1β), which was consistent with Neu1 activation and enhanced inflammatory responses. Histopathological examination further revealed progressive vascular congestion and epithelial injury, suggesting an association between Neu1–ZBP1 signaling and virus-induced tissue damage. Collectively, our findings suggest that Neu1 may contribute to ZBP1-associated PANoptosis during BCoV infection and link sialic acid metabolism to nucleic acid-sensing and inflammatory cell death responses. These results provide mechanistic insight into BCoV pathogenesis and suggest Neu1 as a potential target for future studies aimed at controlling coronavirus infections in cattle and possibly other species.

**Supplementary Information:**

The online version contains supplementary material available at 10.1186/s13567-026-01729-7.

## Introduction

Bovine coronavirus (BCoV) is a highly contagious pathogen that causes severe diarrhea in calves and adult cattle and is frequently accompanied by respiratory symptoms, fever, and decreased milk production, leading to major economic losses in the cattle industry. BCoV belongs to the family *Coronaviridae*, subfamily *Orthocoronavirinae*, and genus *Betacoronavirus*, which also includes human coronaviruses such as severe acute respiratory syndrome coronavirus 2 (SARS-CoV-2) and Middle East respiratory syndrome coronavirus (MERS-CoV) [1]. First identified in the Americas in 1972 and isolated in 1984, BCoV has since been reported in multiple countries and remains prevalent in China [2]. Importantly, its host range is not limited to cattle, as infections have also been detected in sheep, goats [3], deer, and other ruminants [4], which can act as reservoirs and facilitate viral spread. BCoV targets both the gastrointestinal and respiratory systems, causing intestinal villus atrophy with crypt hyperplasia, as well as severe necrosis of alveolar epithelial cells [5].

BCoV is an enveloped, positive-sense single-stranded RNA virus approximately 100 nm in diameter with an ~31 kb genome—the largest known RNA viral genome [6, 7]. Its structural proteins include spike (S), nucleocapsid (N), and membrane (M) proteins. Among these proteins, the S protein is critical for host cell entry and the induction of immune responses [8]. Recent studies have shown that BCoV hijacks host galactose-specific lectins to facilitate spike protein recognition of 9-*O*-acetyl-*N*-acetylneuraminic acid (Neu5,9Ac2) residues on the cell surface, highlighting the importance of sialic acid in viral attachment and entry [9, 10].

Neuraminidase 1 (Neu1), a key regulator of sialic acid metabolism, has attracted increasing attention. Neu1 is widely expressed in microorganisms and mammals, where it regulates cell differentiation, proliferation, and apoptosis [11, 12]. Interestingly, Neu1 functions in a context-dependent manner: High Neu1 expression in hepatocellular carcinoma or cardiomyocytes is correlated with poor prognosis [13–15], whereas elevated Neu1 levels in prostate or colorectal cancer are associated with the induction of programmed cell death [16]. This functional duality suggests that Neu1 may serve as a critical molecular switch in diverse pathological processes and as a potential biomarker for disease progression. However, the precise regulatory role of Neu1 in coronavirus infection and its relationship with host cell physiology remain poorly understood.

Coronavirus infections are frequently accompanied by dysregulated inflammation, often manifesting as cytokine storms [17]. These hyperinflammatory responses are closely linked to multiple forms of programmed cell death (PCD), including apoptosis, pyroptosis, and necroptosis. In 2019, Kanneganti and colleagues introduced the concept of PANoptosis, an integrated form of cell death mediated by the assembly of a multiprotein PANoptosome complex, which orchestrates crosstalk between apoptotic, pyroptotic, and necroptotic pathways [18]. PANoptosis is pathogen-specific and shaped by the host genetic context [19]. Notably, during coronavirus replication, Z-form nucleic acids (Z-NAs), which are recognized by the sensor protein Z-DNA binding protein 1 (ZBP1), can be generated [20]. This recognition triggers PANoptosome assembly and PANoptosis. While this mechanism has been established in other viral infections, it has not been investigated in the context of BCoV.

Given that coronaviruses frequently exploit sialylated glycoconjugates for host attachment and that sialic acid remodeling can shape inflammatory microenvironments, Neu1-mediated desialylation may influence host responses during coronavirus infection. Moreover, ZBP1 is a central sensor that links coronavirus-derived Z-form nucleic acids to PANoptosis; however, how ZBP1-associated PANoptosis is modulated by host metabolic factors remains unclear. These considerations led us to hypothesize that Neu1-dependent sialic acid metabolism may be functionally connected to ZBP1–PANoptosome signaling during BCoV infection. Therefore, we investigated whether Neu1 is induced by BCoV and whether Neu1 is related to ZBP1-associated PANoptosis and viral replication.

Therefore, this study aimed to determine whether Neu1 modulates PANoptosis during BCoV infection through regulation of the ZBP1–PANoptosome axis. To explore this question in vivo, we employed genetically stable inbred BALB/c mice, which are laboratory-accessible strains that are susceptible to BCoV and develop detectable viral replication and inflammatory responses [21]. This laboratory-accessible model enables reproducible investigations of BCoV pathogenesis and host immunity. By examining this previously unexplored pathway, our results may provide new mechanistic insight into coronavirus-induced cell death and inform future efforts to develop targeted strategies for preventing and controlling BCoV infection.

## Materials and methods

### Ethics statement

All experimental procedures involving animals were conducted in accordance with the guidelines for the care and use of laboratory animals. Specific pathogen-free (SPF) male BALB/c mice were obtained from the Laboratory Animal Center of Yanbian University. The experimental protocol was reviewed and approved by the Animal Ethics Committee of Yanbian University (approval no. YD20250827010).

### Cell culture and virus infection

The bovine coronavirus strain (BCoV-YBYJ) was maintained at the Preventive Veterinary Medicine Laboratory, College of Agriculture, Yanbian University. The virus was stably passaged ten times in Madin–Darby bovine kidney (MDBK) cells in vitro prior to use in subsequent experiments, with a final viral titer of 2 × 10^7^ TCID_50_/mL. MDBK cells were purchased from Zhejiang Baidibio Co., Ltd. and cultured in Roswell Park Memorial Institute (RPMI)-1640 medium (Baidi, Zhejiang) supplemented with 10% fetal bovine serum (FBS; Baidi, Zhejiang). For viral infection, the required volume of viral suspension was calculated according to the desired multiplicity of infection (MOI). The cells were incubated with the virus at 37 °C in 5% CO_2_ for 30 min to allow adsorption, after which fresh RPMI-1640 medium was added for further culture.

### Mouse infection

BALB/c mice that were 28 days old were divided into mock-infected control and BCoV-infected groups at two time points, 4 and 7 days post-infection (dpi) (*n* = 3 per group per time point; total *n* = 12). BCoV-infected mice were orally inoculated with 1 mL of BCoV suspension (2 × 10^7^ TCID_50_/mL), whereas mock controls received 1 mL of sterile 1× phosphate-buffered saline (PBS) by oral gavage and were processed in parallel at the same time points. The mice were euthanized at 4 or 7 dpi, and the tissues were collected for histopathology, viral detection, and serological analyses.

### Histopathological analysis

Normal and pathological tissues or organs were fixed in 4% paraformaldehyde at room temperature for 24 h, followed by standard procedures of dehydration, clearing, paraffin infiltration, and embedding. The paraffin blocks were sectioned into thin slices via a microtome, and the sections were subjected to hematoxylin and eosin (H&E) staining. After deparaffinization with xylene and rehydration through a graded ethanol series, the slides were mounted and examined under a light microscope.

### MDBK-ZBP1-knockdown cell construction

The single guide (sgRNA) sequence (5′-CCTGGGGATGAAGACAGCGA-3′) was designed on the basis of the bovine *ZBP1* reference sequence (GenBank accession no. XM_015474249), cloned, and inserted into the lentiCRISPR-v2 vector, which was then transfected into MDBK cells via Lipofectamine 2000 (Thermo Fisher Scientific, USA). The transduced cells were cultured in Dulbecco’s modified Eagle medium (DMEM) supplemented with 10% FBS and selected with 5 μg/mL puromycin. After expansion of the puromycin-resistant clones, *ZBP1* knockdown was confirmed by western blotting, and the resulting cell line was designated MDBK^ZBP1kd^.

### Small interfering RNA (siRNA) transfection

Small interfering RNAs (siRNAs) specifically targeting the bovine *Neu1* gene (GenBank accession no. NM_001083642.1) were designed and synthesized. MDBK cells were seeded at an appropriate density and transfected with siRNAs via Lipofectamine 2000 (Thermo Fisher Scientific, USA) according to the manufacturer’s instructions. After 24 h of incubation under standard culture conditions, the cells were infected with BCoV at a multiplicity of infection (MOI) of 1. Following infection, the cells were maintained in complete medium and harvested at 48 h post-infection for subsequent assays, including quantitative polymerase chain reaction (qPCR) and western blot analysis, to evaluate gene silencing efficiency, viral replication, and downstream signaling pathways.

### RNA isolation and real-time PCR

Total RNA was isolated via TRIzol reagent (GenStar, Beijing), and reverse transcription was conducted with StarScript III RT SuperMix (GenStar, Beijing) to synthesize first-strand complementary DNA (cDNA) for qPCR. The qPCR assays (20 µL) included 0.5 µL of each primer (Additional file 1), 10 µL of 2 × RealStar Universal SYBR qPCR mix (GenStar, Beijing), 1 µL of cDNA template, and 8 µL of nuclease-free water. The cycling conditions were as follows: 95 °C for 2 min, followed by 40 cycles of 95 °C for 10 s and 60 °C for 15 s.

### Western blotting

Total cellular proteins were extracted on ice via NP-40 lysis buffer supplemented with a protease inhibitor cocktail at a ratio of 100:1. Following centrifugation at 14 000 rpm for 10 min, the supernatants were subjected to sodium dodecyl sulfate–polyacrylamide gel electrophoresis (SDS‒PAGE) and subsequently transferred onto 0.45 μm polyvinylidene difluoride (PVDF) membranes (Merck, Germany). Immunoblotting was performed with the appropriate primary antibodies, including anti-ZBP1 (13285–1, Proteintech, Wuhan), anti-Neu1 (67032–1, Proteintech, Wuhan), anti-RIPK3 (17563–1, Proteintech, Wuhan), anti-Caspase-1 (P29466, Abways, Shanghai), anti-Caspase-8 (CY5038, Abways, Shanghai), anti-phospho-MLKL (CY5146, Abways, Shanghai), anti-RIPK1 (WL04522, Wanlei, Shenyang), anti-GSDMD (WL05686, Wanlei, Shenyang), anti-ASC (WL02462, Wanlei, Shenyang), anti-β-actin (AB0011, Abways, Shanghai), and anti-GAPDH (AB0036, Abways, Shanghai) antibodies. All the western blot results were quantified by densitometric analysis using ImageJ (Fiji, 1.53t, USA).

### Immunofluorescence

The cells or tissue sections were rinsed three times with PBS and subsequently fixed at room temperature with 4% paraformaldehyde. The cell membranes were permeabilized with 0.1% Triton X-100, followed by blocking with 5% bovine serum albumin (BSA). Immunofluorescence labeling was then performed with the indicated primary antibodies, and fluorescence localization was visualized under a fluorescence microscope (Nikon, C-SHG1, Japan). The antibodies used included ZBP1 (13285–1, Proteintech, Wuhan), Neu1 (67032–1, Proteintech, Wuhan), GSDMD (WL05686, Wanlei, Shenyang), RIPK3 (17563–1, Proteintech, Wuhan), dsRNA (85780–2-RR, Proteintech, Wuhan), and Caspase-8 (sc-73526, Santa Cruz, USA). Dihydroethidium (DHE; G1904, Servicebio, Wuhan) was used as the fluorescent probe for detection. The secondary antibodies used for immunofluorescence were Cy3-conjugated goat anti-mouse IgG (A0521; Beyotime, Beijing) and FITC-conjugated goat anti-rabbit IgG (A0562; Beyotime, Beijing). Nuclei were counterstained with DAPI (C1006; Beyotime, Beijing). All the immunofluorescence experiments were performed independently three times. For each condition in each experiment, images were acquired via identical acquisition settings from at least three random non-overlapping fields. Image quantification was carried out via ImageJ.

### Flow cytometry

For apoptosis analysis, treated cells were detached via trypsin digestion, resuspended in complete culture medium, and collected via centrifugation. The pellets were washed three times with 1× PBS and subsequently resuspended in 1× Annexin V-PE binding buffer (0.1 M HEPES, 1.4 M NaCl, and 25 mM CaCl_2_ solution; Servicebio, Wuhan) at the appropriate cell density. Annexin V-PE (G1512, Servicebio, Wuhan) was added to the cell suspension and incubated for 8 min at room temperature in the dark, followed by staining with 7-AAD on ice for 5 min. After supplementation with 1× binding buffer, the samples were immediately subjected to flow cytometric analysis via a Beckman flow cytometer. Data were acquired and analyzed with FlowJo (version 10; FlowJo Software, USA) to determine the proportions of apoptotic and necrotic cells.

### Sialic acid and IL-1β detection

Sialic acid levels in cells and serum were determined via a commercial sialic acid assay kit (A036-2; Jiancheng, Jiangsu). The cellular sialic acid content was measured following standard protein extraction procedures, whereas the serum samples were directly subjected to analysis. Briefly, sialic acid detection reagent was mixed with the samples in a 96-well plate and incubated at 37 °C for 15 min. Absorbance was then measured at 340 nm via a microplate reader, and concentrations were calculated according to the manufacturer’s instructions and the corresponding formula.

The concentration of IL-1β in mouse serum was determined using a mouse IL-1β enzyme-linked immunosorbent assay (ELISA) kit (KE10003, Proteintech, Wuhan). Total protein was extracted following standard procedures, and protein concentrations were quantified via the bicinchoninic acid (BCA) assay. A standard curve was generated using the kit-provided standards, and the assay was performed according to the manufacturer’s instructions, following the sequential steps of sample loading, washing, and color development. The absorbance was measured at 450 nm via a microplate reader, and the IL-1β concentration was calculated on the basis of the standard curve and the corresponding formula.

### Drug treatment and gene overexpression

The cells were treated with *N‐*benzyloxycarbonyl‐Val‐Ala‐Asp(OMe) fluoromethyl ketone (Z-VAD-FMK; CAS no. 161401–82-7, TargetMol, Shanghai), necrostatin-1 (Nec-1; CAS no. 4311–88-0, TargetMol, Shanghai), and 2,3-dehydro-2-deoxy-*N*-acetylneuraminic acid (Neu5Ac2en; CAS no. 24967–27-9, Macklin, Shanghai). The optimal working concentrations of all the compounds were determined via CCK-8 assays (APExBIO, USA) and subsequently subjected to western blot analysis. The *Neu1* sequence (GenBank accession no. NM_001083642.1) was used for construct design, and the predicted signal peptide was removed before cloning into the pcDNA3.1 vector. Transfection was performed with Lipofectamine 2000, followed by western blot detection.

### Protein‒protein docking and molecular dynamics simulation

Molecular docking between bovine-derived ZBP1 and Neu1 was performed on the basis of structure-guided docking, and the resulting complex was used as the initial structure for molecular dynamics (MD) simulations. All MD simulations were carried out using GROMACS (version 2022, the Netherlands). The force field parameters were generated using the pdb2gmx utility with the AMBER99SB force field, and additional parameters were obtained from the AutoFF web server when necessary. Bond constraints involving hydrogen atoms were applied using the LINCS algorithm.

The simulation protocol consisted of energy minimization with positional restraints applied to the solute, followed by equilibration of solvent molecules and counterions. Subsequently, a 100 ns production molecular dynamics (MD) simulation was performed under constant-number-of-particles, pressure, and temperature (NPT) conditions at 310 K and 1 atm. Throughout the simulation, structural and dynamic properties, including root mean square deviation (RMSD), root mean square fluctuation (RMSF), hydrogen bond formation, radius of gyration (Rg), solvent-accessible surface area (SASA), and binding free energy (calculated using the molecular mechanics/Poisson–Boltzmann surface area [MM/PBSA] method), were analyzed.

#### GST pull-down

We constructed the prokaryotic expression plasmids pGEX-4 T-1-ZBP1-GST and pET32a-Neu1-6 × His on the basis of the *ZBP1* sequence (GenBank accession no. XM_015474249) and the *Neu1* sequence (GenBank accession no. NM_001083642.1) obtained from the National Center for Biotechnology Information (NCBI). The empty pGEX-4 T-1-GST vector was used as a negative control. Each plasmid was transformed into *E. coli* BL21 competent cells (Coolaber, Beijing), and protein expression was induced with isopropyl-β-d-thiogalactopyranoside (IPTG; TaKaRa, Japan) at low temperature followed by purification. GST pull-down assays were performed using GST-tag magnetic beads (P2258, Beyotime, Beijing), and the proteins were incubated with the beads overnight at 4 °C. The beads were then boiled in 2× protein loading buffer, and the eluates were subjected to western blot analysis. The antibodies used were anti-His (no. T0009, Affinity, Jiangsu) and anti-GST (no. T0007, Affinity, Jiangsu).

#### Co-immunoprecipitation (Co-IP)

Total cellular proteins were extracted via NP-40 lysis buffer. After preclearing with protein A/G agarose beads (P2055, Beyotime, Beijing) for 1 h at room temperature, the supernatants were incubated overnight at 4 °C with specific antibodies, followed by incubation with protein A/G agarose beads for 1 h at room temperature. The immunocomplexes were washed four to five times, eluted by boiling in 2× protein loading buffer for 5 min, and analyzed by western blotting. The antibodies used included anti-ZBP1 (sc-271483, Santa Cruz, USA), anti-Neu1 (67032–1, Proteintech, Wuhan), and an anti-mouse IgG isotype control (AB0032, Abways, Shanghai).

### Statistical analysis

All the statistical analyses were performed via GraphPad Prism (version 9.5.1; GraphPad Software). The data are presented as the means ± standard deviations (SDs) from at least three independent biological replicates. Multiple group comparisons were analyzed via one-way or two-way analysis of variance (ANOVA). A *P* value < 0.05 was considered statistically significant. The levels of significance are indicated as ^*^P < 0.05, ^**^P < 0.01, ^***^P < 0.001, ^****^P < 0.0001, and not significant (ns).

## Results

### Serological and histopathological changes in BCoV-infected BALB/c mice

**Figure 1:**
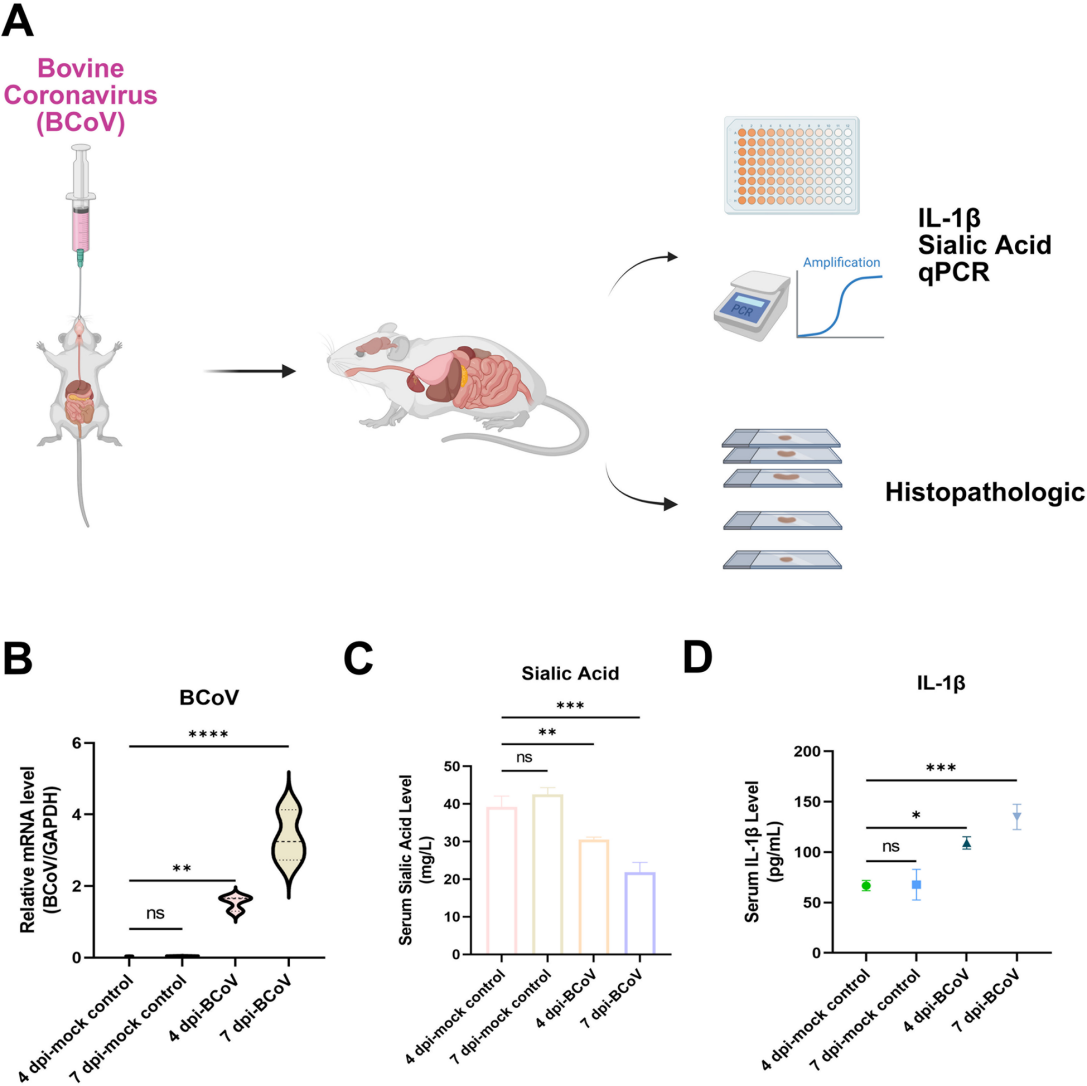
**Serological and qPCR analysis of BALB/c mice following BCoV infection.** **A** Schematic of the experimental design: 28-day-old BALB/c mice were orally inoculated with BCoV (2 × 10^7^ TCID_50_/mL, 1 mL) and sacrificed at 4 dpi and 7 dpi for histopathological examination, IL-1β and sialic acid measurements, and viral detection. **B** Relative messenger RNA (mRNA) levels of BCoV in BALB/c colons determined by qPCR (normalized to GAPDH). **C** Serum sialic acid levels were measured via a commercial assay kit. **D** Serum IL-1β levels were measured via ELISA. The data are presented as the means ± SDs (*n* = 3). Statistical significance: ^**^P < 0.01, ^***^P < 0.001, ^****^P < 0.0001, ns, not significant.

BALB/c mice were used as an in vivo model for experimental BCoV infection (Figure 1A). Tissues exhibiting potential pathological changes were subsequently subjected to histopathological evaluation and viral replication analysis. Owing to viral tissue tropism, colonic tissues were examined first, where viral titers were found to increase progressively, indicating active viral replication (Figure 1B). In addition, BCoV infection in BALB/c mice was associated with a decrease in serum sialic acid concentrations (Figure 1C) and a progressive increase in IL-1β levels (Figure 1D), which was consistent with altered sialic acid metabolism and enhanced inflammatory responses.

Gross pathological examination was performed to assess tissue changes in BCoV-infected BALB/c mice. At 7 days post-infection, overt congestion was observed in the brain, whereas only mild macroscopic changes were noted in the colon compared with those in the control group. To further investigate these observations, hematoxylin and eosin (H&E) staining was conducted to evaluate histopathological alterations.

**Figure 2:**
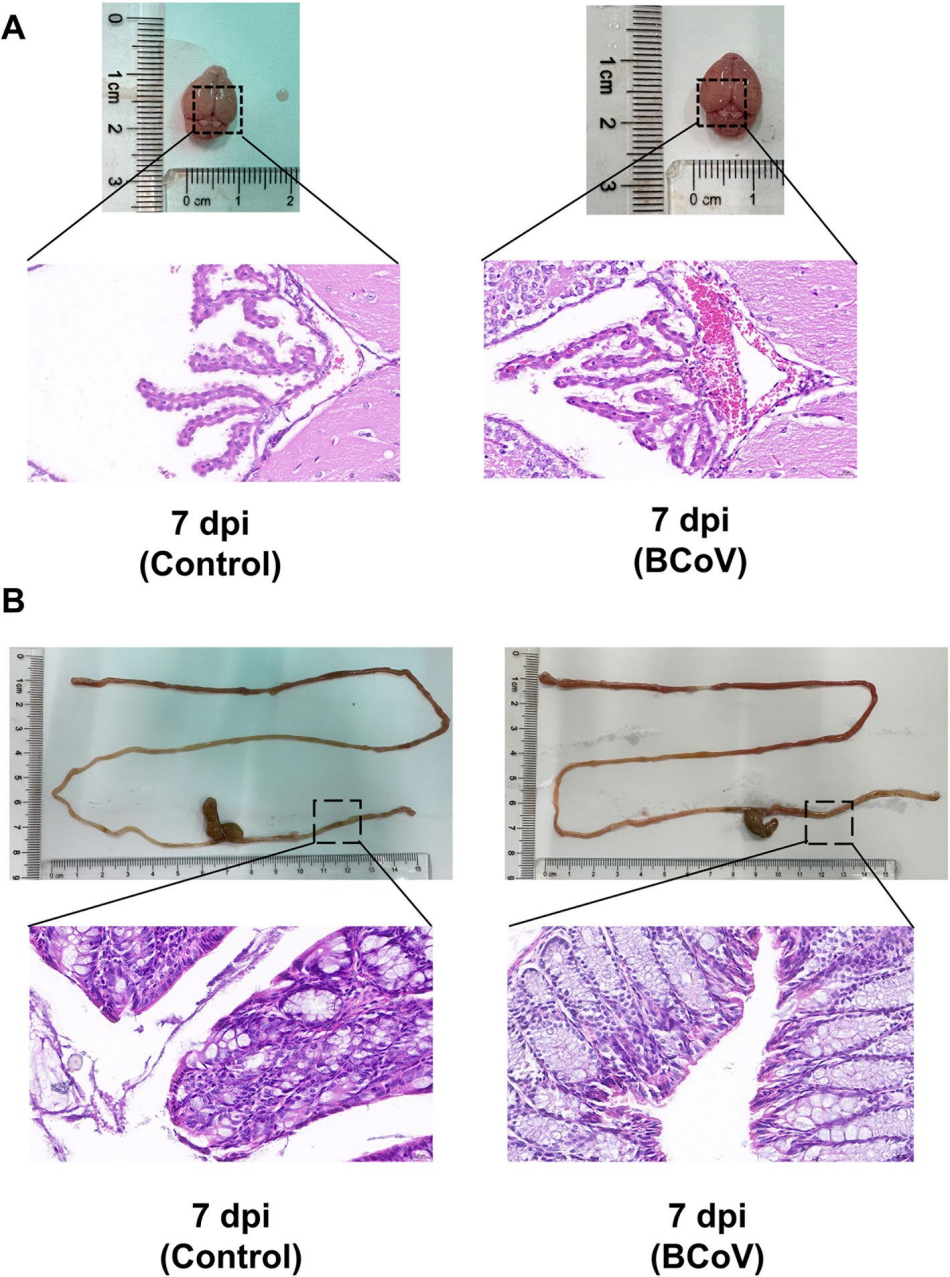
**Histopathological alterations in the brain and colonic tissues of BALB/c mice following BCoV infection.** **A** Brain: Compared with time-matched controls, BCoV-infected mice presented marked vascular congestion, erythrocyte extravasation, and choroid plexus disruption. **B** Compared with control infection, BCoV infection caused glandular disruption, goblet cell reduction, mucosal atrophy, and interstitial edema.

In brain sections, mock-infected controls presented a normal choroid plexus architecture with intact vessels and no obvious erythrocyte extravasation. In contrast, BCoV-infected mice at 7 dpi presented pronounced vascular congestion, erythrocyte extravasation, and disruption of surrounding tissue structures, which was consistent with inflammatory injury in the choroid plexus (Figure 2A).

In the colonic tissues, the time-matched control mice at 7 dpi presented well-organized glands, intact mucosal architecture, and abundant goblet cells. In contrast, the BCoV-infected group exhibited glandular disruption, reduced goblet cells, mucosal atrophy, and interstitial edema (Figure 2B). Together with the brain findings, these results indicate that BCoV infection is associated with inflammatory and structural injury in both the brain and colon at this stage of infection.

### *Neu1* and *ZBP1* show increased expression and spatial proximity in vivo during BCoV infection

**Figure 3:**
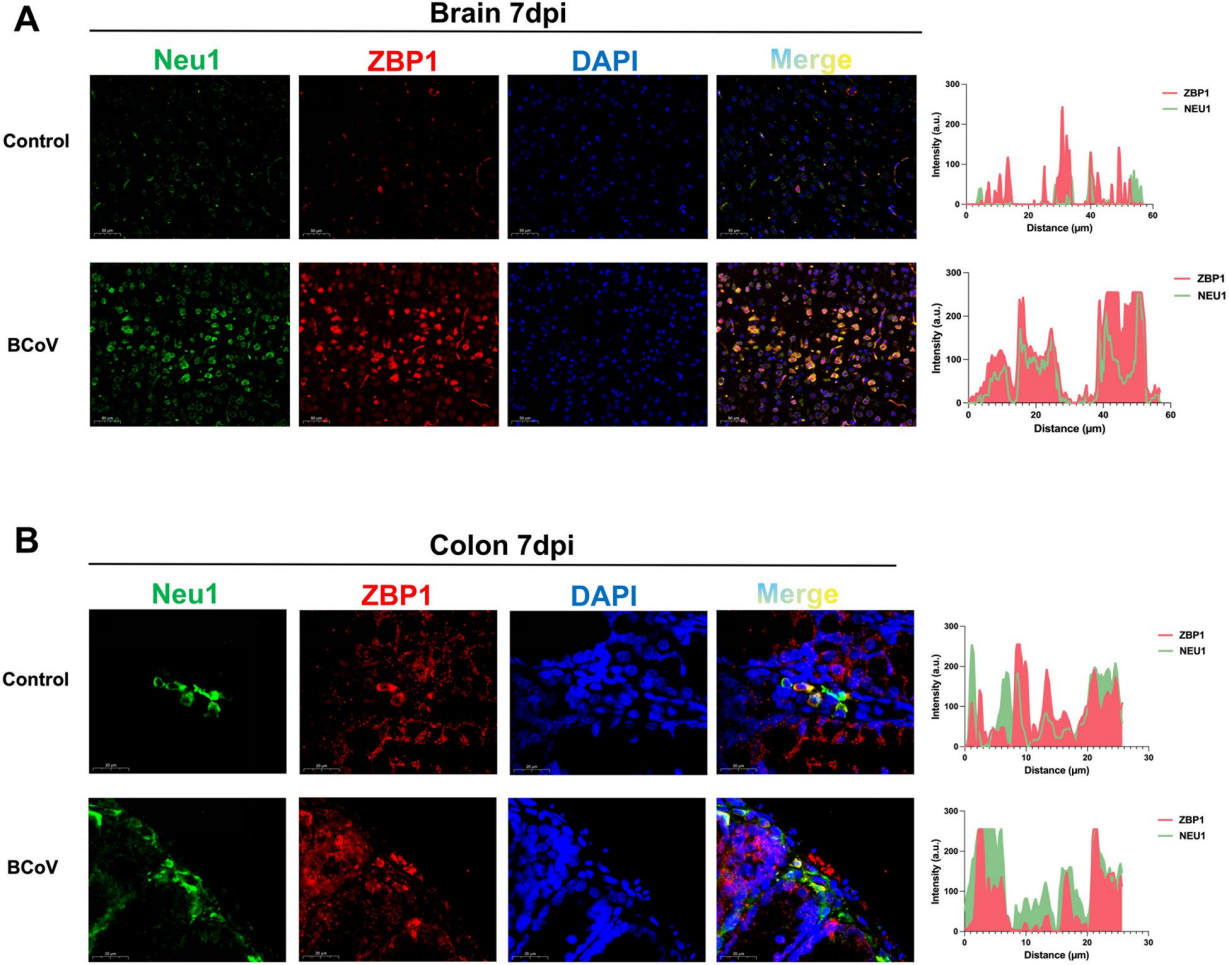
**Neu1 and ZBP1 immunofluorescence in the brain and colon at 7 dpi.** **A**, **B** Representative immunofluorescence images of Neu1 (green) and ZBP1 (red) in brain (**A**) and colon (**B**) sections from time-matched mock controls and BCoV-infected BALB/c mice at 7 days post-infection. The control tissues presented weak basal signals, whereas the infected tissues presented increased Neu1 and ZBP1 fluorescence with areas of partial overlap in the merged images. The fluorescence intensity profiles are shown on the right.

To determine whether BCoV infection affects the expression and spatial distribution of ZBP1 and Neu1 in BALB/c mice, we performed immunofluorescence costaining of brain and colonic sections from time-matched mock controls and BCoV-infected mice at 7 days post-infection. In control brains, ZBP1 and Neu1 exhibited only weak basal fluorescence. In infected brains, both signals were markedly increased, and the merged images revealed areas of partial overlap, indicating close spatial proximity between ZBP1 and Neu1 at this stage of infection (Figure 3A). In the colon, ZBP1 and Neu1 fluorescence was also greater in infected mice than in control mice, although the overall signal intensity appeared lower than that observed in the brain, possibly reflecting tissue-specific differences or infection-associated structural alterations (Figure 3B).

Quantitative fluorescence intensity analysis supported these observations, showing that both Neu1 and ZBP1 signals were significantly greater in infected mice than in control mice. In addition, the partial overlap of Neu1 and ZBP1 staining in brain and colonic tissues indicates close spatial associations in vivo during BCoV infection. Together, these results suggest that BCoV infection is accompanied by increased Neu1 and ZBP1 expression and spatial proximity within affected tissues, which is consistent with a potential Neu1–ZBP1 relationship that may contribute to host responses and disease progression.

### BCoV infection activates the ZBP1–PANoptosome complex and *Neu1* in host cells

**Figure 4:**
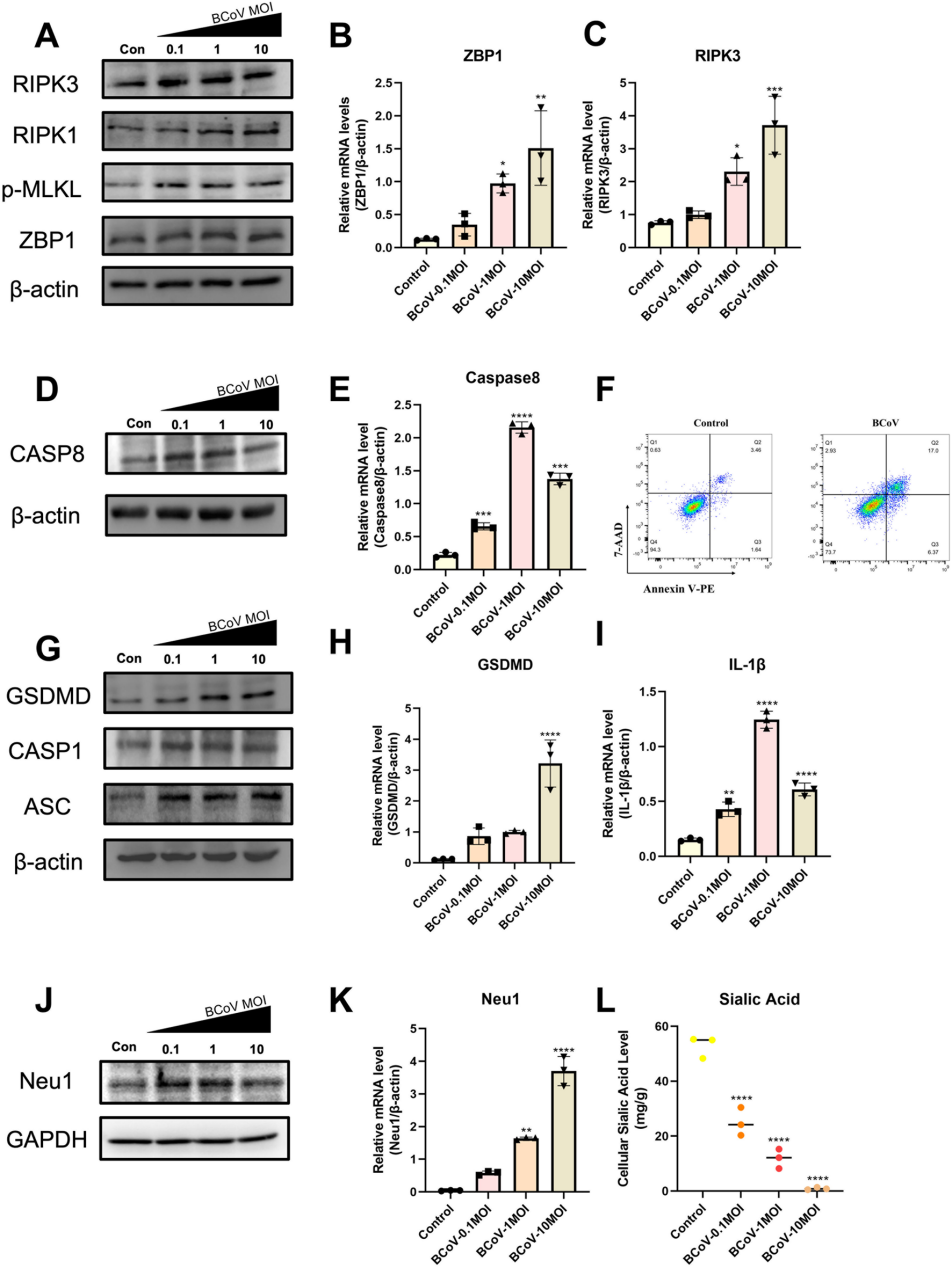
**BCoV infection induces the expression of PANoptosis-related genes in MDBK cells.** **A** Western blot analysis of RIPK3, RIPK1, p-MLKL, and ZBP1 protein levels. **B**, **C** Relative mRNA levels of *ZBP1* (**B**) and *RIPK3* (**C**) determined by qPCR. **D**, **E** Protein (**D**) and mRNA (**E**) expression of CASP8. **F** Flow cytometric analysis of apoptosis via Annexin V-PE/7-AAD staining in control and BCoV-infected cells. **G**, **H** Protein (**G**) and mRNA (**H**) expression of GSDMD, CASP1, and ASC. **I** Relative mRNA expression of *IL-1β*. **J**, **K** Protein (**J**) and mRNA (**K**) expression of Neu1. **L** Cellular sialic acid levels, which decreased in a dose-dependent manner upon BCoV infection. Quantitative densitometry of the western blot results (normalized to β-actin/GAPDH) is shown in Additional file 2. The data are presented as the means ± SDs (*n* = 3). Statistical significance: ^*^P < 0.05, ^**^P < 0.01, ^***^P < 0.001, ^****^P < 0.0001.

To further investigate the pathogenic mechanisms of BCoV, we inoculated MDBK host cells with BCoV at MOIs of 0.01, 0.1, and 1 and collected the cells at 24 h post-infection for analysis. After infecting cells with different multiplicities of infection, markers associated with necrosis, including receptor-interacting protein kinase 3 (RIPK3), RIPK1, phosphorylated mixed lineage kinase domain-like protein (MLKL), and ZBP1, tended to increase in expression as the viral titer increased (Figure 4A). qRT‒PCR further confirmed the transcriptional levels of the necroptosis markers *ZBP1* and *RIPK3*, which was consistent with the western blot results (Figures 4B, C). These findings suggest that BCoV infection significantly activates the necroptosis pathway involving ZBP1.

PANoptosis involves multiple programmed cell death mechanisms, including CASP8, Gasdermin D (GSDMD), CASP1, and apoptosis-associated speck-like protein containing a CARD (ASC), which constitute the PANoptosome. Therefore, it is essential to examine other processes of programmed cell death (PCD). Among the apoptosis-related genes, CASP8 was upregulated at early stages with increasing infection doses but tended to be downregulated at high viral titers (Figure 4D). Quantitative reverse transcription (qRT)‒PCR validated the expression of *CASP8* (Figure 4E), which was in line with the western blot results. Additionally, we assessed apoptosis levels in host cells via Annexin V-PE/7-AAD double staining, which revealed a clear trend toward apoptosis following BCoV infection, indicating that BCoV induces apoptosis in host cells (Figure 4F).

We further explored the expression of pyroptosis-related genes within the ZBP1–PANoptosome complex. The results revealed significant upregulation of pyroptosis markers, including GSDMD, CASP1, and ASC, as the viral titer increased (Figure 4G). The transcriptional levels of *GSDMD* also confirmed these findings. Furthermore, we measured the transcriptional levels of *IL-1β* after infection and detected significant upregulation at an MOI of 1 (Figures 4H–I), further suggesting that BCoV infection triggers pyroptosis in host cells. These PANoptosis-related markers showed dose-dependent induction, with a peak at the intermediate MOI, followed by a modest decline at the highest MOI, yet remained elevated compared with those of the controls.

To confirm whether BCoV infection activates Neu1, we performed western blot and qRT‒PCR analyses. Similar to the gene expression trends within the ZBP1–PANoptosome complex, Neu1 expression was upregulated with increasing viral titers (Figures 4J, K). We continued to assess sialic acid levels within the cells and observed an inverse correlation between sialic acid content and viral titer (Figure 4L), indicating that BCoV infection leads to desialylation and an increase in intracellular Neu1 protein expression.

### Knockdown of *Neu1* or *ZBP1* suppresses BCoV replication in host cells

**Figure 5:**
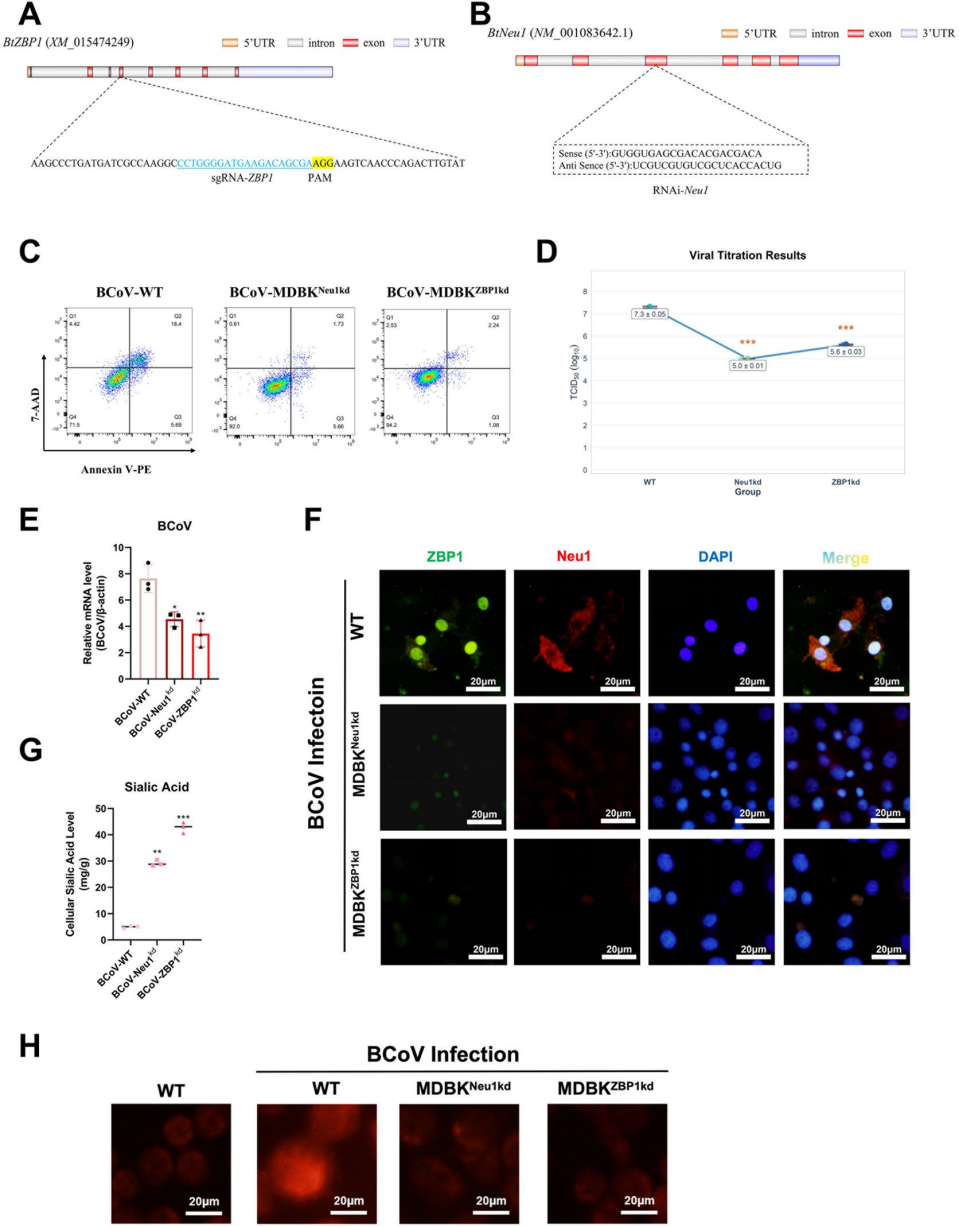
**The interaction between Neu1 and ZBP1 regulates apoptosis and sialic acid levels in BCoV-infected MDBK cells.** **A**, **B** Schematic representation and validation of stable MDBK^Neu1kd^ and MDBK^ZBP1kd^ cell lines. **C** Apoptosis analysis of WT, MDBK^Neu1kd^, and MDBK^ZBP1kd^ cells following BCoV infection via Annexin V-PE/7-AAD flow cytometry. **D**, **E** Viral titers determined via the Reed–Muench method (**D**) and relative BCoV mRNA expression levels (**E**). **F** Immunofluorescence localization of Neu1 (red) and ZBP1 (green) in WT, MDBK^Neu1kd^, and MDBK^ZBP1kd^ cells. Nuclei were stained with DAPI (blue), and merged signals (yellow) indicate colocalization. **G** Cellular sialic acid levels significantly decreased in both MDBK^Neu1kd^ and MDBK^ZBP1kd^ cells. **H** Immunofluorescence analysis further demonstrated that *Neu1* or *ZBP1* knockdown markedly reduced the fluorescence signal intensity during BCoV infection. The data are presented as the means ± SDs (*n* = 3). The quantification of fluorescence intensity is shown in Additional file 4. Statistical significance: ^*^P < 0.05, ^**^P < 0.01, ^***^P < 0.001.

On the basis of the above findings, a *ZBP1*-targeting CRISPR–Cas9 knockdown plasmid and a *Neu1*-specific siRNA sequence were designed and transfected into MDBK cells via standard transfection protocols. To assess apoptosis induced by BCoV, Annexin V-PE/7-AAD staining was performed in MDBK wild-type (MDBK^WT^), *Neu1*-knockdown (MDBK^Neu1kd^), and *ZBP1*-knockdown (MDBK^ZBP1kd^) cell lines. The results revealed that, compared with the wild-type treatment, the knockdown of *Neu1* or *ZBP1* significantly reduced the proportions of apoptotic and necrotic cells (Figure 5C). Furthermore, viral titers were determined across the three cell lines via the Reed–Muench method, which revealed decreased viral titers in both knockdown cell lines. Consistent with these findings, qPCR analysis confirmed that silencing *Neu1* or *ZBP1* markedly suppressed BCoV replication in MDBK cells (Figures 5D-E).

Immunofluorescence staining revealed that Neu1 (red) and ZBP1 (green) were colocalized in MDBK^WT^ cells following BCoV infection, as evidenced by the prominent yellow signals in the merged images. In contrast, in MDBK^Neu1kd^ cells, ZBP1 signals were markedly diminished, with a concomitant reduction in the degree of colocalization. Similarly, in MDBK^ZBP1kd^ cells, Neu1 expression was reduced, and the merged fluorescence intensity was attenuated, further confirming a decrease in Neu1–ZBP1 colocalization (Figure 5F). These results indicate that the interaction between Neu1 and ZBP1 is dependent on the expression of both proteins and is significantly impaired upon the knockdown of either gene. Similarly, we observed that sialic acid levels tended to increase with decreasing ZBP1 and Neu1 levels (Figure 5G). For the assessment of oxidative stress, intracellular superoxide levels were measured via dihydroethidium (DHE) staining. BCoV-infected MDBK^WT^ cells presented marked accumulation of reactive oxygen species (ROS), whereas *Neu1*- and *ZBP1*-knockdown cells presented reduced ROS levels. These results suggest that silencing *Neu1* or *ZBP1* alleviates BCoV-induced oxidative stress and cellular injury (Figure 5H).

To further validate the interaction between Neu1 and ZBP1, cells were treated with the Neu1 inhibitor Neu5Ac2en, as well as the ZBP1 inhibitors Z-VAD-FMK and Nec-1, while bovine *Neu1* overexpression was used as a positive control. Western blot analysis of treated and transfected cells revealed that the inhibition of Neu1 also suppressed ZBP1 expression, whereas Z-VAD-FMK and Nec-1 treatment reduced Neu1 expression. These findings provide additional evidence for a synergistic regulatory relationship between Neu1 and ZBP1 (Additional file 3).

### *Neu1* knockdown mitigates PANoptosis triggered by BCoV in host cells

**Figure 6:**
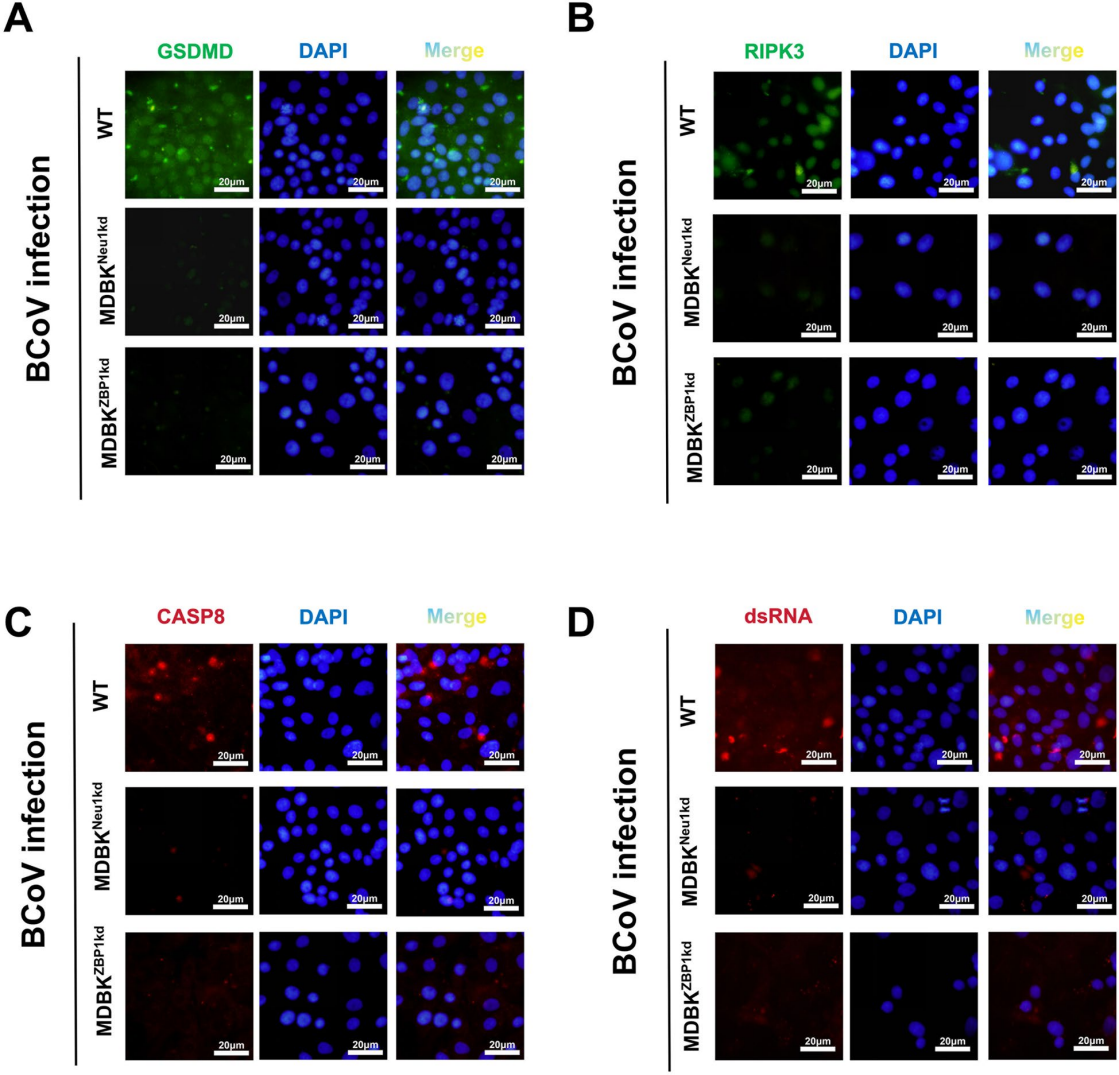
**Effects of Neu1 or ZBP1 knockdown on PANoptosis-related gene expression during BCoV infection. A**–**D** Immunofluorescence detection of GSDMD (**A**, green), RIPK3 (**B**, green), CASP8 (**C**, red), and dsRNA (**D**, red) in WT, MDBK^Neu1kd^, and MDBK^ZBP1kd^ cells. Nuclei were stained with DAPI (blue), and merged images are shown. Strong fluorescence signals were observed in WT cells, whereas signal intensities were markedly reduced in *Neu1*- or -deficient cells. The quantification of fluorescence intensity is shown in Additional file 4.

Building upon these observations, we next performed immunofluorescence assays to evaluate the expression of representative effector proteins of the ZBP1–PANoptosome complex, including GSDMD (Figure 6A), RIPK3 (Figure 6B), CASP8 (Figure 6C), and dsRNA (Figure 6D). In MDBK^WT^ cells, all three proteins presented strong fluorescence signals, indicative of active PANoptosis signaling upon BCoV infection. In contrast, their expression was consistently reduced in the MDBK^Neu1kd^ and MDBK^ZBP1kd^ cell lines, suggesting that the knockdown of either *Neu1* or *ZBP1* markedly impaired the activation of PANoptosis effector pathways. In addition, a dsRNA (J2) antibody was used to confirm viral infection. Strong dsRNA signals were readily detected in WT cells, whereas the fluorescence intensity was markedly reduced in MDBK^Neu1kd^ and MDBK^ZBP1kd^ cells (Figure 6D). These results indicate that the knockdown of *Neu1* or *ZBP1* decreases the susceptibility of MDBK cells to BCoV infection.

**Figure 7:**
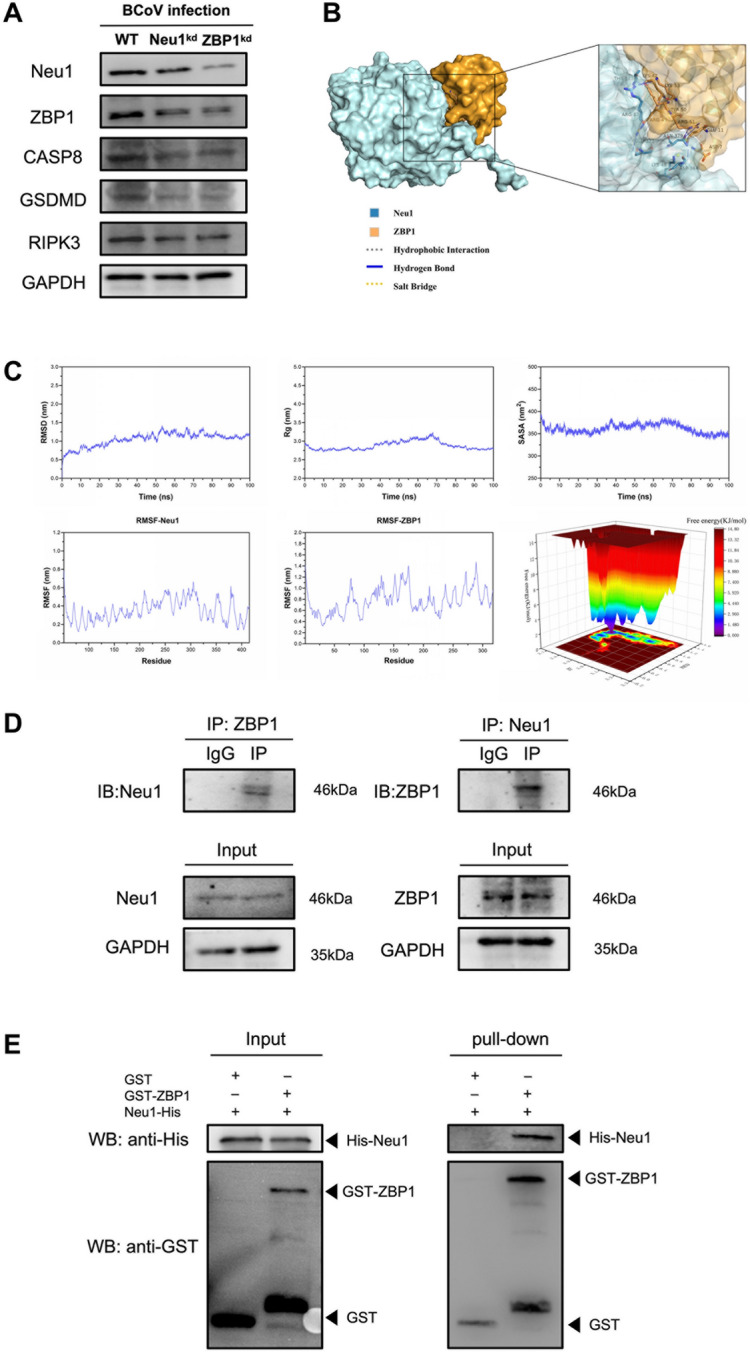
**Validation of the Neu1–ZBP1 interaction and molecular dynamics simulation analysis.** **A** Western blot analysis of Neu1, ZBP1, CASP8, GSDMD, and RIPK3 expression in WT, MDBK^Neu1kd^, and MDBK^ZBP1kd^ cells. The results were consistent with the immunofluorescence data, indicating that the knockdown of *Neu1* or *ZBP1* downregulated the expression of PANoptosis effector proteins. **B** Protein–protein docking model of Neu1 and ZBP1. The predicted docking interface was visualized via PyMOL, and the magnified view highlights hydrogen-bond interactions between the two proteins. **C** Molecular dynamics simulations of the Neu1–ZBP1 complex performed with GROMACS 2022 for 100 ns. Analyses of RMSD, RMSF, Rg, HBonds, SASA, and free energy landscape (FEL) demonstrated that the Neu1–ZBP1 complex exhibited stable binding with favorable hydrogen-bond interactions. **D** Co-IP assays demonstrating the interaction between Neu1 and ZBP1. **E** GST pull-down assay showing the in vitro interaction between GST-ZBP1 and His-Neu1. Quantitative densitometry of the western blot results (normalized to that of GAPDH) is shown in Additional file 5.

Western blotting was then conducted to corroborate the immunofluorescence data, revealing expression patterns that were fully consistent with the observed staining results. Specifically, the levels of CASP8, GSDMD, and RIPK3 were substantially lower in both knockdown cell lines than in WT cells, confirming that *Neu1* depletion attenuates the activation of ZBP1–PANoptosome-associated effector molecules. Collectively, these results support the conclusion that *Neu1* knockdown mitigates BCoV-induced PANoptosis, at least in part, by downregulating the expression of key effector proteins within the ZBP1–PANoptosome complex (Figure 7A).

To further examine the potential Neu1–ZBP1 interaction, protein–protein docking was performed in PyMOL (Figure 7B). The predicted model revealed several hydrogen-bond contacts at the Neu1–ZBP1 interface, suggesting structural complementarity. We then evaluated the behavior of this predicted complex via molecular dynamics simulations via GROMACS 2022 (Figure 7C). RMSD, RMSF, radius of gyration, and hydrogen-bond analyses indicated that the complex adopted a relatively stable configuration over the simulation time, with limited fluctuations and sustained interfacial contacts.

To provide experimental support, we next assessed the Neu1–ZBP1 interaction via both Co-IP and GST pull-down. Co-IP revealed that Neu1 and ZBP1 coprecipitated in BCoV-infected cells, whereas no signal was detected in the IgG negative control (Figure 7D), indicating a specific association in the cellular context. To minimize potential intracellular confounders, we purified bacterially expressed GST-ZBP1 and His-Neu1 and performed an in vitro GST pull-down assay with GST alone as a control. His-Neu1 was detected in the pull-down fraction only in the presence of GST-ZBP1 but not with GST alone (Figure 7E).

Taken together, the computational and biochemical results support a specific Neu1–ZBP1 association and are consistent with a direct interaction between the two proteins during BCoV infection.

## Discussion

Programmed cell death (PCD) involves a complex network of mechanisms that collectively maintain host defense and homeostasis. Among these, PANoptosis has recently been defined as an integrated form of cell death that simultaneously encompasses apoptosis, pyroptosis, and necroptosis. This process is tightly orchestrated by nucleic acid sensors such as ZBP1, which recognizes intracellular Z-form nucleic acids and subsequently triggers the assembly of the PANoptosome complex. The PANoptosome functions as a multiprotein signaling platform, integrating distinct death pathways into a unified system [22]. This integration ensures not only the elimination of virus-infected cells, thereby limiting viral dissemination, but also enhances antiviral immunity through the maturation and release of proinflammatory cytokines.

**Figure 8:**
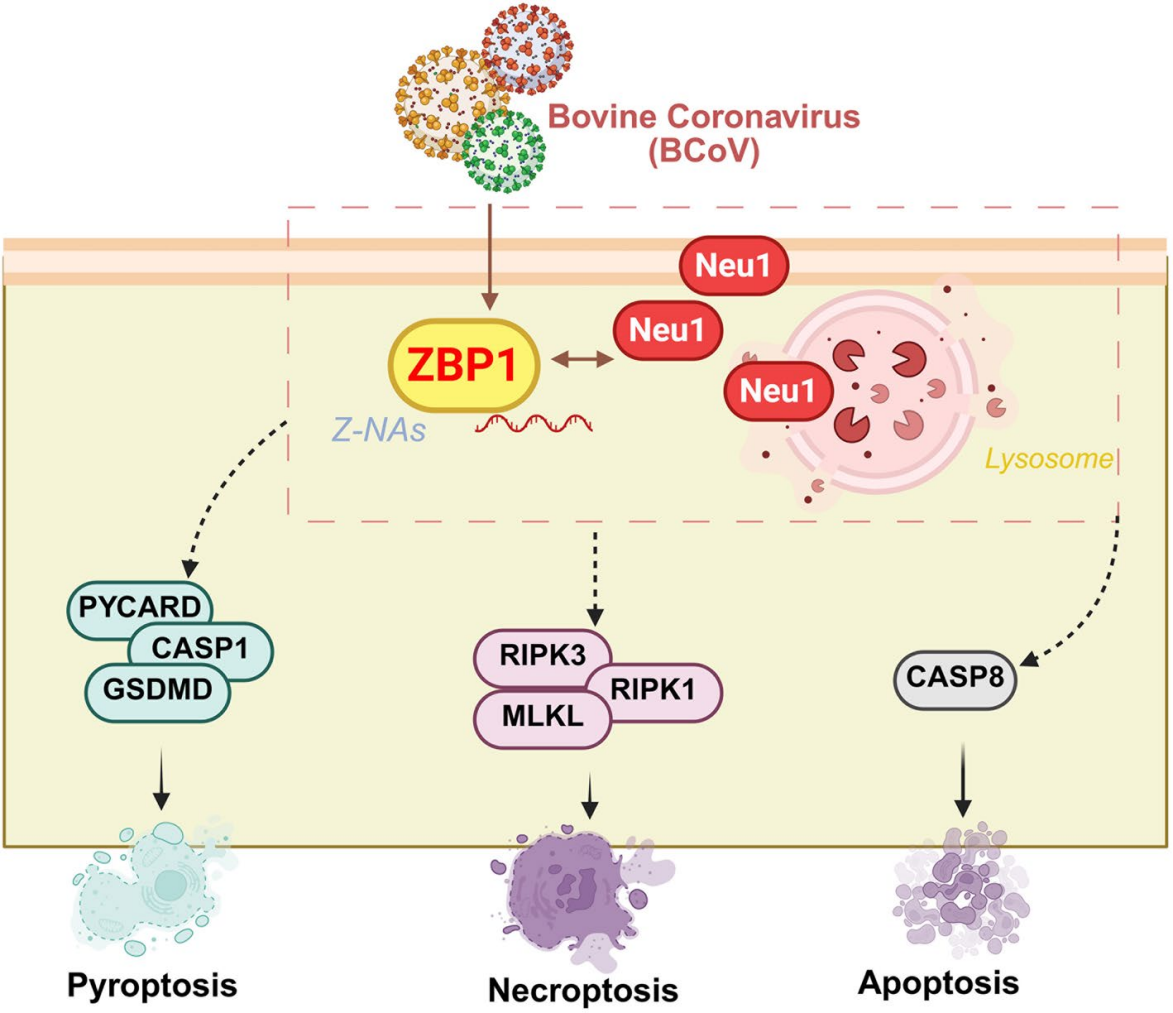
**Proposed model of Neu1- and ZBP1-associated PANoptosis during BCoV infection.** Following BCoV entry, Neu1 activity is induced at the plasma membrane and/or lysosomal compartments and is accompanied by a specific association with the nucleic acid sensor ZBP1. ZBP1 recognizes intracellular Z-NAs generated during infection and is linked to PANoptosome-associated signaling. Downstream, PANoptosis-related effectors, including pyroptosis-associated components (PYCARD–CASP1–GSDMD), necroptosis-associated factors (RIPK3–RIPK1–MLKL), and apoptosis-related CASP8, are involved. Together, these pathways are concomitantly activated during BCoV infection and may contribute to inflammatory cell death responses and changes in viral replication.

The role of ZBP1 in viral infection is exemplified by influenza A virus, where Z-RNA within viral ribonucleoproteins (vRNPs) is detected by ZBP1 [23, 24]. Through RHIM-dependent interactions, ZBP1 recruits RIPK3, CASP8, and NLRP3 to assemble the PANoptosome [25, 26], which in turn coordinates GSDMD-mediated pyroptosis, Caspase-driven apoptosis, and MLKL-dependent necroptosis [27–29]. This cooperative network constitutes a stringent antiviral defense (Figure 8). However, excessive or dysregulated PANoptosis can result in uncontrolled inflammation, tissue damage, and worsened disease outcomes. Similar phenomena have been observed in infections with herpes simplex virus 1 (HSV-1) [30, 31], Zika virus [32], and coronaviruses such as SARS-CoV-2 and murine hepatitis virus (MHV), where ZBP1-dependent PANoptosis exacerbates pathology by driving IL-1β/IL-18-mediated cytokine storms and MLKL-induced membrane rupture [33–35].

While the contribution of ZBP1 to PANoptosis is well established, much less is known about how its activity is modulated by host factors. Here, we identified Neu1 as a previously unrecognized regulator of ZBP1. Neu1, a lysosomal sialidase, not only participates in sialic acid catabolism but also fulfills diverse roles critical for cellular homeostasis [36]. By hydrolyzing terminal sialic acid residues on glycoproteins and glycolipids [37], Neu1 regulates the conformation and activation of receptors such as epidermal growth factor receptor (EGFR) [38], Toll-like receptors [39], and the insulin receptor [40], thereby influencing signaling pathways that govern proliferation, immunity, and metabolism. In immune cells, Neu1 fine-tunes receptor sialylation to modulate cytokine secretion [41], phagocytosis [42], and T cell activation [43, 44]. In addition to these physiological functions, Neu1 has also been implicated in viral pathogenesis. Current evidence remains limited, with studies focusing mainly on influenza A virus and hepatitis B virus. During influenza A infection, Neu1 acts as a key host determinant for viral entry, while viral neuraminidase further exploits host sialic acids to facilitate replication and assembly [45]. In hepatitis B virus infection, Neu1 activation enhances the phosphorylation of downstream NF-κB, thereby promoting inflammatory responses [46]. These findings suggest that Neu1 contributes to viral assembly and release in both RNA and DNA viruses and is involved in the induction of programmed cell death. In summary, Neu1 is a multifaceted regulator that integrates glycosylation remodeling with receptor signaling, immune regulation, and viral pathogenesis and, as demonstrated here, extends to the control of ZBP1-driven PANoptosis.

The relationship between Neu1 and ZBP1 is supported by several complementary observations in our study. In vitro, *Neu1* knockdown was accompanied by reduced ZBP1 levels and attenuated PANoptosis-related signaling, whereas Neu1 stimulation was associated with increased ZBP1-related responses and downstream effector expression. In vivo, Neu1 and ZBP1 signals increased after BCoV infection and showed partial spatial overlap in brain and colonic tissues, which coincided with decreased serum sialic acid and elevated IL-1β. Co-immunoprecipitation, GST pull-down, and molecular dynamics analyses supported a specific physical association between Neu1 and ZBP1 and suggested a potentially stable interaction interface. Taken together, these findings indicate that Neu1 is linked to ZBP1-associated PANoptosis during BCoV infection and may influence related inflammatory and viral replication outcomes, although the precise mechanistic roles require further investigation.

The potential relevance of these findings may extend beyond BCoV. Neu1 and ZBP1 are conserved in many mammals and have been implicated in host responses to diverse viral infections. On this basis, it is possible that Neu1–ZBP1-associated signaling could also operate during infection with other betacoronaviruses, although this remains to be tested directly. If such conservation is confirmed, modulation of this pathway might influence PANoptosis-related inflammation and tissue injury in a broader coronavirus context. From a translational perspective, Neu1 represents a host factor whose role in viral replication and inflammatory cell death responses warrants further investigation. Whether targeting Neu1 can achieve antiviral and anti-inflammatory benefits will require dedicated validation in additional models and species.

In summary, our data suggest that Neu1 is associated with ZBP1-related PANoptosis during BCoV infection. The observed alterations in Neu1 activity, ZBP1–PANoptosome signaling, and spatial proximity in infected tissues support a potential Neu1–ZBP1 relationship linking sialic acid metabolism to nucleic acid-sensing pathways and inflammatory cell death responses. Although further work is needed to define the precise mechanistic and in vivo relevance of this interaction, these findings provide new insight into host responses to BCoV and indicate that Neu1 may represent a pathway of interest for future antiviral studies.

## Supplementary Information

**Additional file 1.** **Primers used in this study.**

**Additional file 2.** **Densitometric analysis of the western blot results is shown in Figure**  4. The band intensities were quantified via ImageJ and normalized to those of β-actin/GAPDH. The data are presented as the means ± SDs (n = 3). Statistical significance: *P < 0.05, **P < 0.01, ***P < 0.001.

**Additional file 3.** **The expression levels of Neu1 and ZBP1 were examined after drug treatment.** (A–B) A CCK-8 assay was used to determine the optimal drug concentrations. Combined treatment with Z-VAD-FMK and Nec-1 had the optimal effect at 50 μM, whereas Neu5Ac2en treatment was optimal at 100 μM. (C) Western blot analysis of cellular protein expression following drug treatment, with pcDNA3.1-Neu1 serving as a positive control. (D) Densitometric analysis of the western blot results. The data are presented as the means ± SDs (*n* = 3). Statistical significance: *P < 0.05, **P < 0.01, ***P < 0.001.

**Additional file 4.** **Fluorescence intensity quantification corresponding to Figures** 4–5**.** The signals from ≥ 3 random nonoverlapping fields per condition in three independent experiments were measured via ImageJ. The data are presented as the means ± SDs (*n* = 3). Statistical significance: *P < 0.05, **P < 0.01, **P < 0.001.

**Additional file 5.** **Densitometric analysis of the western blot results is shown in Figure  **7**.** Band intensities were quantified via ImageJ and normalized to those of GAPDH. The data are presented as the means ± SDs (n = 3). Statistical significance: *P < 0.05, **P < 0.01, ***P < 0.001.

## Data Availability

All the data generated or analyzed during this study are provided in this published article.
