# Supplementary material for: Neu1 inhibition restrains BCoV replication and modulates ZBP1-dependent PANoptosis
Source: Vet Res. 2026 Apr 21;57:62. doi: 10.1186/s13567-026-01729-7 (PMC13154460; doi:10.1186/s13567-026-01729-7)
Supplement: Supplementary file 1 — Additional file 1. Primers used in this study. [file 13567_2026_1729_MOESM1_ESM.docx]

Additional file 1. Primers used in this study

| Gene Name | Primers (5’-3’) | Length (bp) |
| --- | --- | --- |
| ZBP1 | F: AGGTCACACAGCTGACCTTGA  R: GGGCCTGTGAGCATAACTCC | 168 |
| Neu1 | F: ATTGTCTTCTTCTCCAACCCAGC  R: GCCTCATCCTTCCCATCCAC | 173 |
| CASP8 | F: AGGACAGTGAGTTGCAGACAT  R: CCTGCTTCCGTGCTATGCTA | 108 |
| GSDMD | F: GCCTTCTGCTTACAGGGCAA  R: AGCGACAGGAAGGTCCTTTG | 164 |
| RIPK3 | F: AGAAGTGCTGGAGCCATGAG  R: AGAAAGGAACTCCTTCACCATGA | 149 |
| β-actin (*Bovine*) | F: GATGATATTGCTGCGCTCGTG  R: CCCACCATTACGCCCTGG | 131 |
| IL-1β | F: CCTCCGACGAGTTTCTGTGT  R: GCTCATGCAGAACACCACTTC | 158 |
| BCoV-1a | F: TTGTGCAAATTACGCGGCAA  R: GAGACGGGCATCTACACTCG | 203 |
| GAPDH (*Mus musculus*) | QM00014S, Beyotime | - |
