# Supplementary figures and images for: Neu1 inhibition restrains BCoV replication and modulates ZBP1-dependent PANoptosis
Source: Vet Res. 2026 Apr 21;57:62. doi: 10.1186/s13567-026-01729-7 (PMC13154460; doi:10.1186/s13567-026-01729-7)

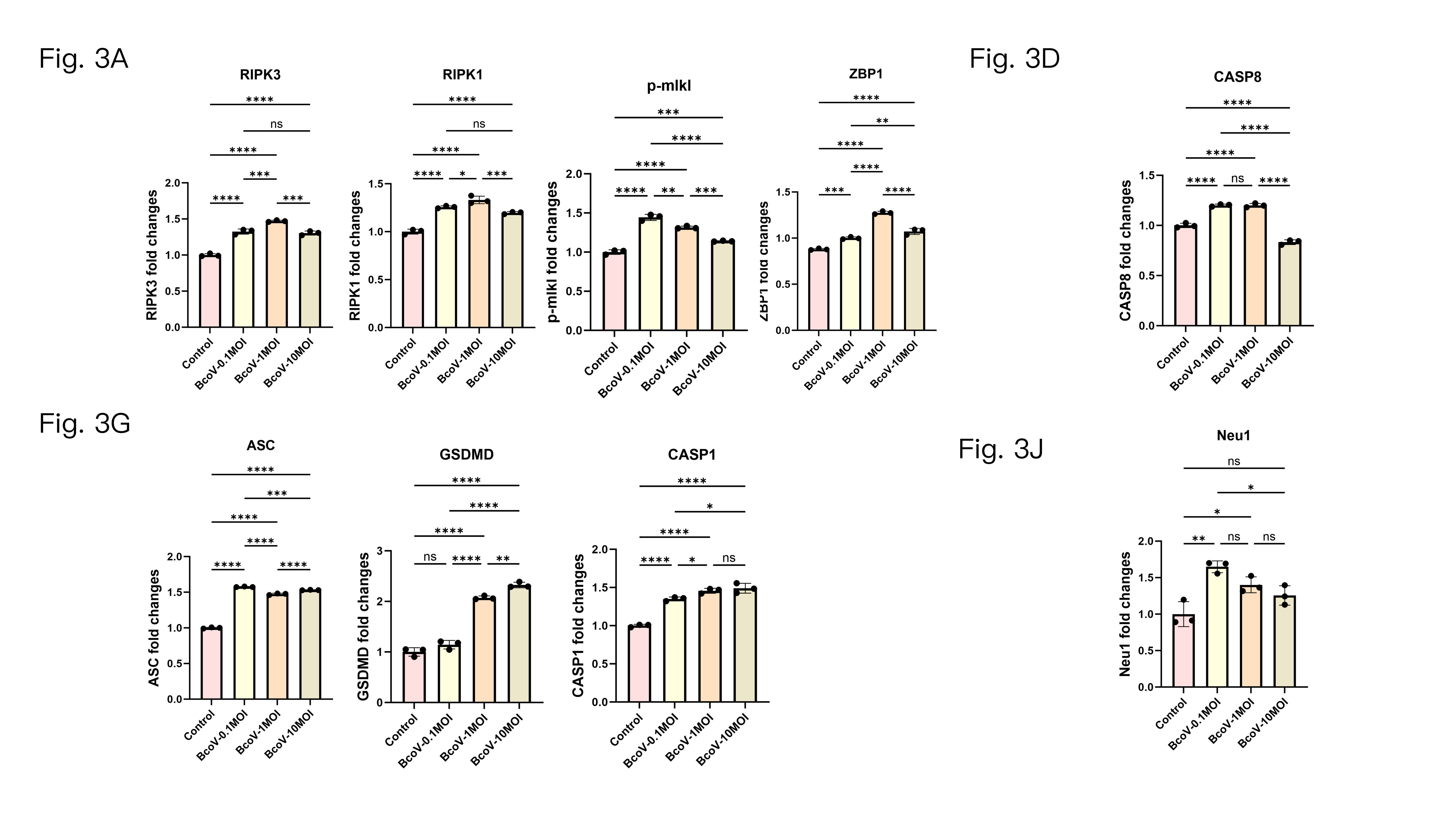

Supplement: Supplementary file 2 — Additional file 2. Densitometric analysis of the western blot results is shown in Figure 4. The band intensities were quantified via ImageJ and normalized to those of β-actin/GAPDH. The data are presented as the means ± SDs (n = 3). Statistical significance: *P < 0.05, **P < 0.01, ***P < 0.001. [file 13567_2026_1729_MOESM2_ESM.tiff]

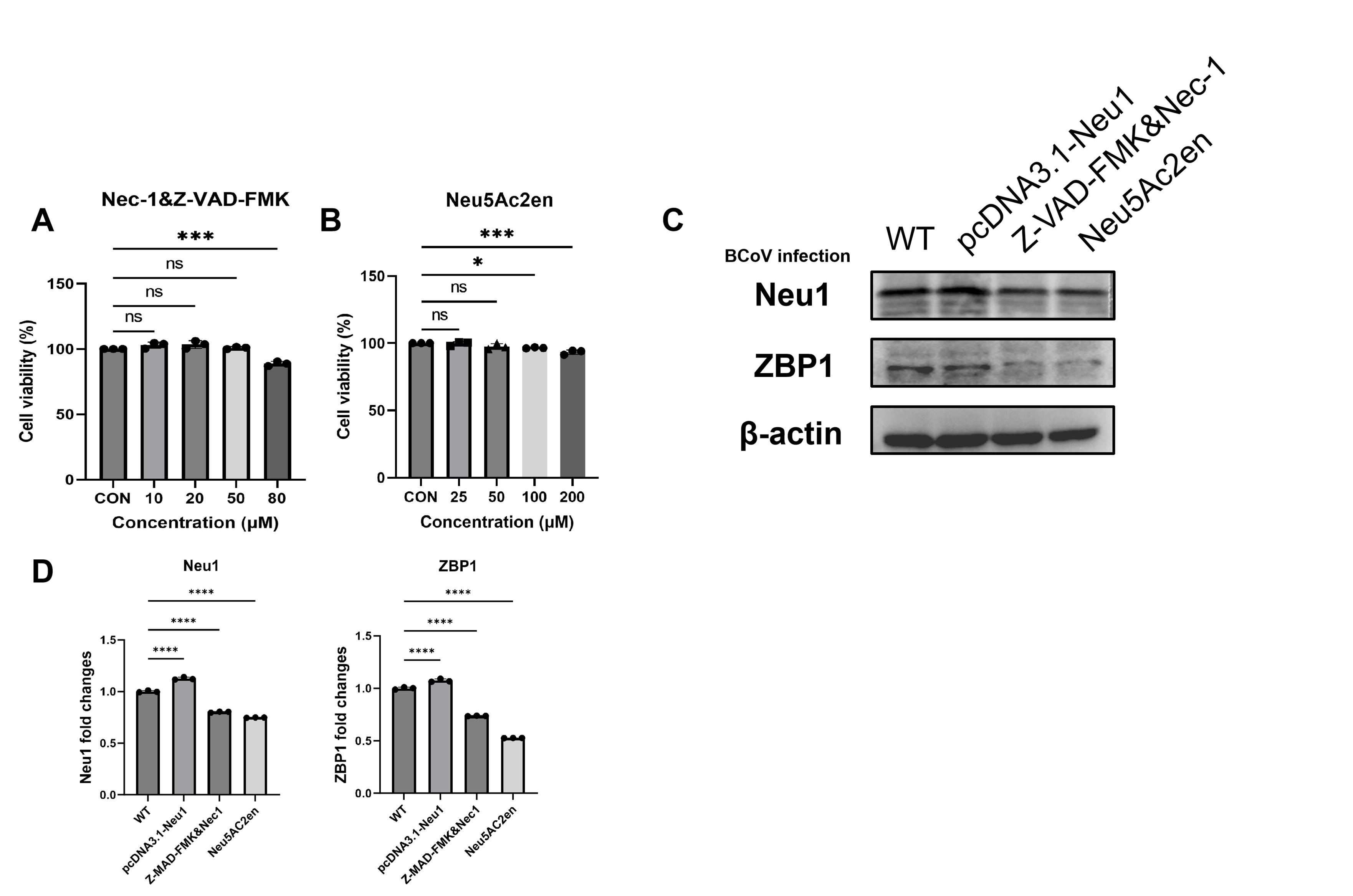

Supplement: Supplementary file 3 — Additional file 3. The expression levels of Neu1 and ZBP1 were examined after drug treatment. (A–B) A CCK-8 assay was used to determine the optimal drug concentrations. Combined treatment with Z-VAD-FMK and Nec-1 had the optimal effect at 50 μM, whereas Neu5Ac2en treatment was optimal at 100 μM. (C) Western blot analysis of cellular protein expression following drug treatment, with pcDNA3.1-Neu1 serving as a positive control. (D) Densitometric analysis of the western blot results. The data are presented as the means ± SDs (n = 3). Statistical significance: *P < 0.05, **P < 0.01, ***P < 0.001. [file 13567_2026_1729_MOESM3_ESM.tiff]

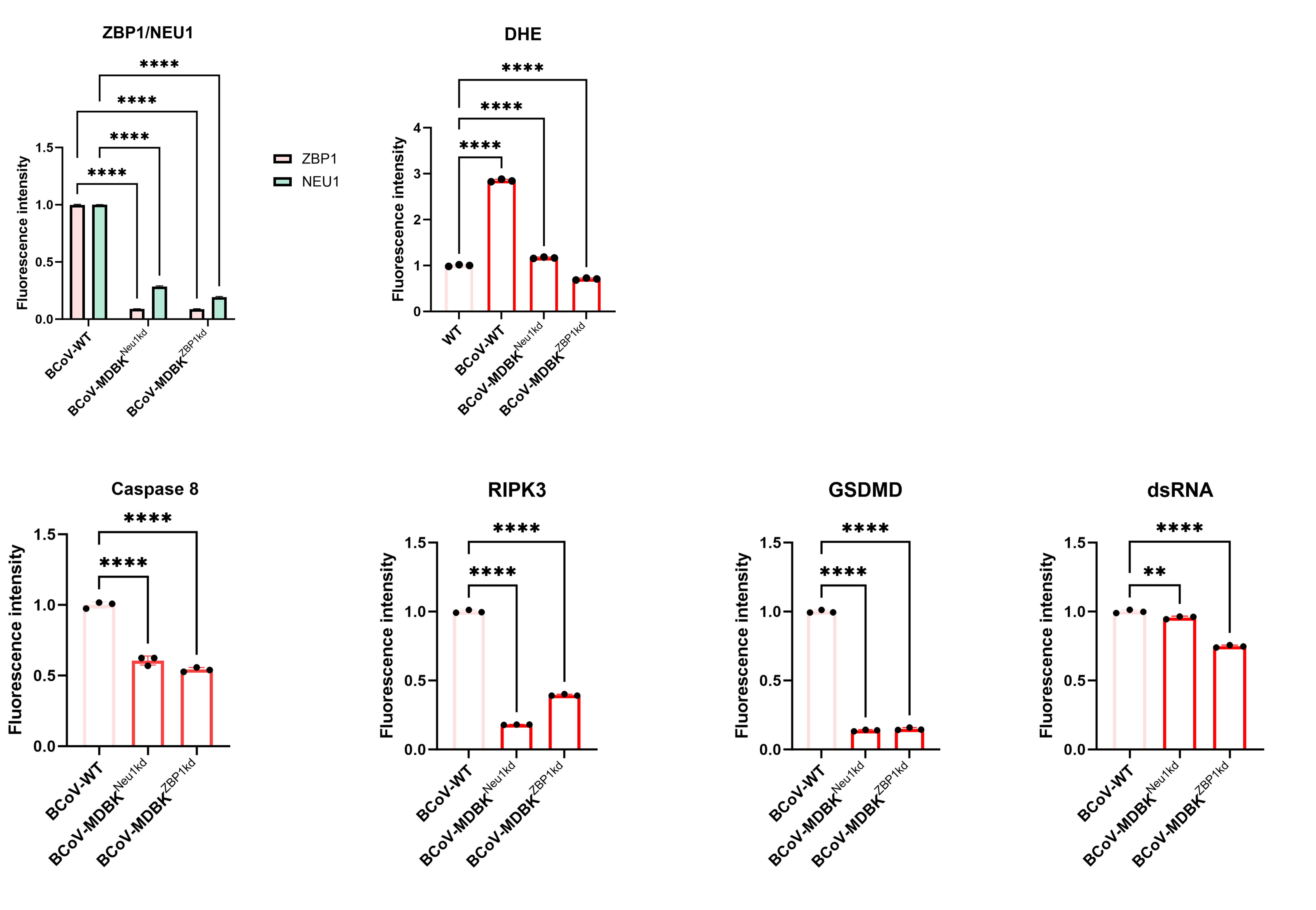

Supplement: Supplementary file 4 — Additional file 4. Fluorescence intensity quantification corresponding to Figures 4–5. The signals from ≥ 3 random nonoverlapping fields per condition in three independent experiments were measured via ImageJ. The data are presented as the means ± SDs (n = 3). Statistical significance: *P < 0.05, **P < 0.01, **P < 0.001. [file 13567_2026_1729_MOESM4_ESM.tiff]

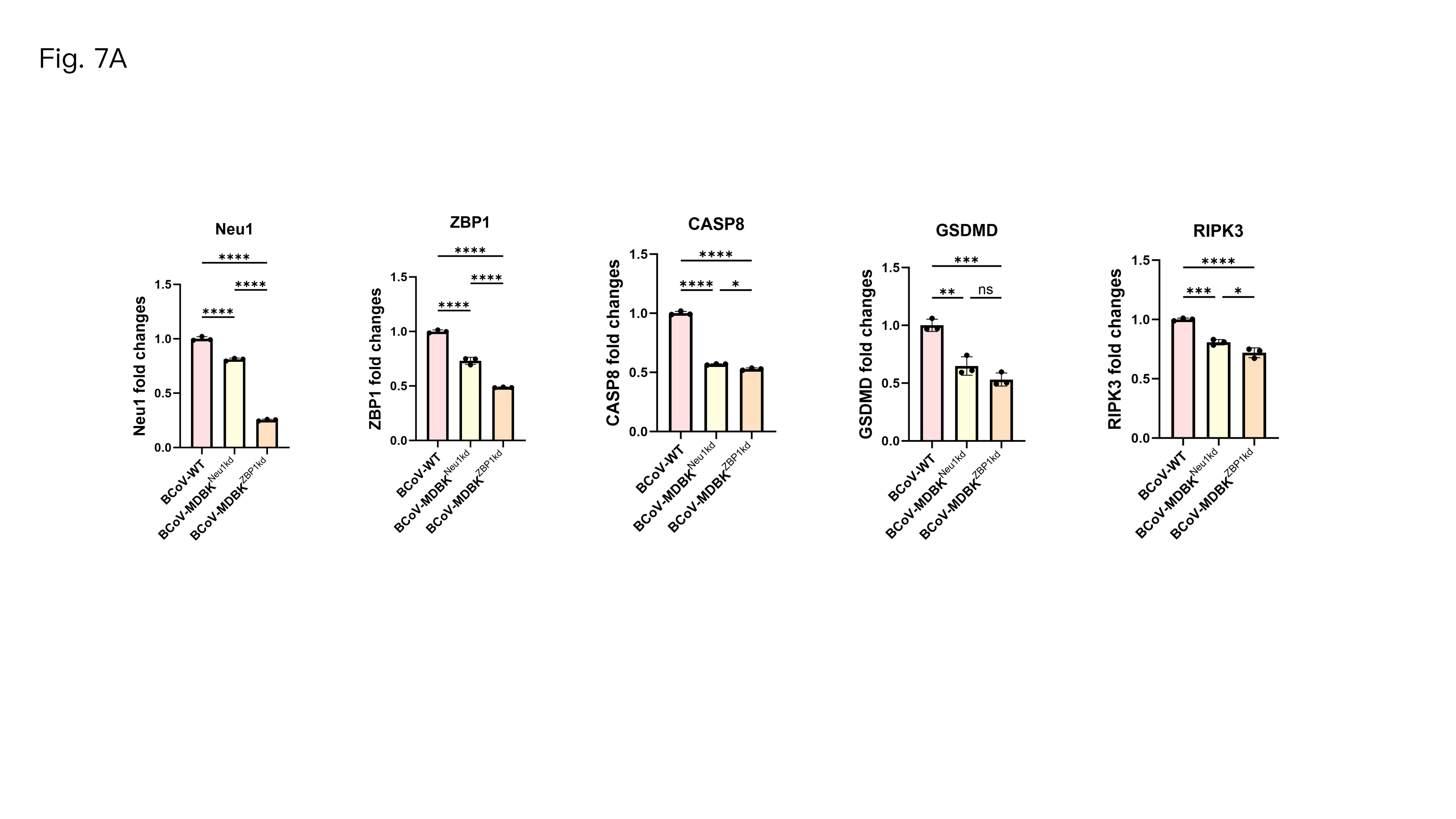

Supplement: Supplementary file 5 — Additional file 5. Densitometric analysis of the western blot results is shown in Figure 7. Band intensities were quantified via ImageJ and normalized to those of GAPDH. The data are presented as the means ± SDs (n = 3). Statistical significance: *P < 0.05, **P < 0.01, ***P < 0.001. [file 13567_2026_1729_MOESM5_ESM.tiff]
